# Dynamic experiments of acid mine drainage with *Rhodopseudomonas spheroides* activated lignite immobilized sulfate-reducing bacteria particles treatment

**DOI:** 10.1038/s41598-022-12897-9

**Published:** 2022-05-24

**Authors:** Junzhen Di, Yiming Ma, Mingjia Wang, Zhenyu Gao, Xiaotain Xu, Yanrong Dong, Saiou Fu, Hanzhe Li

**Affiliations:** 1 College of Civil Engineering, Liaoning Technical University, Fuxin, China; 2 Qingdao Peak Vision Sponge City Construction Engineering Co., Qingdao, China; 3 Qingdao Rongchuang Yacht Industry Investment Co., Qingdao, China

**Keywords:** Biochemistry, Environmental chemistry

## Abstract

Aiming at the problem that the treatment of acid mine drainage (AMD) by sulfate-reducing bacteria (SRB) biological method is susceptible to pH, metal ions, sulfate and carbon source. Lignite immobilized SRB particles (SRB-LP) and *Rhodopseudomonas spheroides* (*R. spheroides*) activated lignite immobilized SRB particles (R-SRB-LP) were prepared using microbial immobilization technology with SRB, *R. spheroides* and lignite as the main substrates. The dynamic experimental columns 1# and 2# were constructed with SRB-LP and R-SRB-LP as fillers, respectively, to investigate the dynamic repair effect of SRB-LP and R-SRB-LP on AMD. The mechanism of AMD treated with R-L-SRB particles was analyzed by scanning electron microscopy (SEM), fourier transform infrared (FTIR) spectrometer and low-temperature nitrogen adsorption. The result showed that the combination of *R. spheroides* and lignite could continuously provide carbon source for SRB, so that the highest removal rates of SO_4_^2−^, Cu^2+^ and Zn^2+^ in AMD by R-SRB-LP were 93.97%, 98.52% and 94.42%, respectively, and the highest pH value was 7.60. The dynamic repair effect of R-SRB-LP on AMD was significantly better than that of SRB-LP. The characterization results indicated that after R-SRB-LP reaction, the functional groups of −OH and large benzene ring structure in lignite were broken, the lignite structure was destroyed, and the specific surface area was 1.58 times larger than before reaction. It illustrated that *R. spheroides* provided carbon source for SRB by degrading lignite. The strong SRB activity in R-SRB-LP, SRB can co-treat AMD with lignite, so that the dynamic treatment effect of R-SRB-LP on AMD is significantly better than that of SRB-LP.

## Introduction

The exploitation of coal resources plays an important role in the industrial development, but a large amount of acid mine drainage (AMD) will be generated during the excavation of coal due to the oxidation of sulfides ores [like pyrite (FeS_2_)]^1^. AMD has a low pH value, high concentrations of sulfate and metal ions. If discharged with improper treatment, it can pose a serious threat to the surrounding environment and human health^2^. So far, AMD treatment is mainly divided into neutralization, adsorption and microbiological methods^3^. The cost of the neutralization method is relatively high, and a large amount of water-containing sludge will be produced during the process^4^. Although the adsorption method has a good removal effect on the metal cation but is not ideal on sulfate.

Microbial treatment method is not only low cost and easy to obtain, but also effective in removing sulfate and heavy metal ions from AMD. In the microbial treatment of AMD, sulfate-reducing bacteria (SRB) as the representative organisms has caused extensive research and application^5^. Muhammad et al.^6^ showed that under suitable conditions, SRB could survive in the environment containing sulfate and various metal ions, and the reduction reaction could reduce 87–100% of iron, lead, copper, zinc and aluminum. Sahinkaya et al.^7^ found that the final sulfate reduction rate could reach 90% in a sulfate-reducing MBR reactor when SRB was inoculated into wastewater with a sulfate concentration of 2000 mg/L and a COD/sulfate of 0.75. SRB is a prokaryote living in anaerobic environment. SRB can use sulfate as the electron acceptor, H_2_ and organic molecule (like alcohol, acetic acid) as the electron donor, and it can reduce sulfate to S^2−^, HS^−^ and H_2_S through alienation reduction effect. The reduced S^2−^ combines with metal ions to form metal sulfide precipitation^8^. SRB is used for treat AMD not only has high reaction efficiency and low reaction cost, but also can recycle valuable metals such as iron and copper in the form of metal sulfide^9^. But the traditional biological method has some problems, such as the strain is easily washed, vulnerable to low pH and metal ions^10^.

Microbial immobilization technology is a method to highly concentrate microbial cells against certain external shocks by using gel interception and biofilm protection^11,12^. The adsorption-embedding method, as a composite immobilization technology, uses the embedding agent as the basic skeleton structure of the immobilized particles, and the particles are filled with the adsorption matrix. It not only effectively solves the problems of low mechanical strength, large mass transfer resistance and low pollutant removal rate of the single immobilization technology, but also provides the adsorption carrier for the bacteria, and improves the mass transfer ability to make the organisms better reproduce^13^. However, the immobilized particles need additional carbon sources to maintain SRB activity. Organic carbon sources such as glucose have high economic costs and are not suitable for large amounts of AMD treatment. Therefore, finding a substrate material that can enhance the pH value of AMD, adsorb heavy metal ions and provide carbon sources for SRB economically and continuously is the key to realize the economic and efficient repair of AMD by SRB.

Lignite reserves are abundant in China, and it is characterized by convenient mining and low cost. Lignite is rich in aromatic benzene ring and functional group-like petroleum structures, such as aliphatic hydrocarbons, aromatic hydrocarbons, carbon and oxygen branched chain^14^. These functional groups can undergo chemical reactions such as ion exchange, coordination and complexation with metal ions to achieve the adsorption effect^15^. Bao et al.^16^ showed that the removal efficiency of Cu^2+^ by lignite reached 67.84 mg/g. M. Ucurum et al.^17^ found that the optimum adsorption capacity of lignite for Pb^2+^ was 29.92 mg/g. Although lignite is economical, easy to obtain and has good adsorption capacity, the carbon source used by SRB is small molecule organic matter such as ethanol and acetic acid. The macromolecular structure contained in lignite cannot be used as carbon source by SRB. The supply of carbon source cannot be realized only using lignite as substrate. However, oxygen bridges and oxygen-containing functional groups in lignite can be metabolized by fermentation bacteria to obtain carbon sources available for microorganisms such as fumaric acid, propionic acid, acetic acid, CO_2_ and H_2_^18^. *Rhodopseudomonas spheroides* (*R. spheroides*) is a photosynthetically fermented bacterium that lives widely in rivers and seas, it is characterized by easy access and high metabolic capacity^19^. Liu et al.^20^ demonstrated that *R. spheroides* have a certain effect of degrading lignite. Xu et al.^21^ showed that *R. spheroides* could degrade aromatic polymers in lignite into low molecular weight substances and other substances. Due to the growth cycle of bacteria, it can maintain activity in a certain time, so *R. spheroides* can continuously decompose lignite, which can meet the sustainability of carbon source supply. At the same time, lignite and *R. spheroides* are easy to obtain, which is in line with the economy of carbon source when dealing with large amounts of AMD. Moreover, lignite itself has good adsorption capacity, which can provide a suitable environment for biological cells to promote the growth of microorganisms and improve the removal efficiency of pollutants.

In view of these phenomenon that during the AMD treated by microbial method microorganisms are easy to lose, the influence of low pH and heavy metals lead to low treatment efficiency, unsustainable and uneconomical use of traditional carbon sources. Based on microbial immobilized technology, SRB, lignite, and *R. spheroides* were used as the main raw materials to make lignite immobilized SRB particles (SRB-LP) and *R. spheroides* activated lignite immobilized SRB particles (R-SRB-LP). A dynamic experiment was set to study the effectiveness of *R. spheroides* decomposing lignite to continuously provide carbon source for SRB and the efficiency of AMD treatment putting lignite as the substrate cooperation with SRB using adsorption-embedding method. The mechanism of R-SRB-LP treating AMD was explored by characterization analysis. Finally, a new method is obtained, which can provide carbon source for SRB economically and continuously, further reduce the influence of AMD on SRB by adsorption capacity and improve the effect of SRB on AMD treatment. This study will provide a new idea for microbial immobilization in AMD treatment.

## Materials and methods

### Experimental materials

#### Test lignite

Samples were taken from Datong, Shanxi Province. The lignite samples were crushed, sieved to a neutral size of 75 μm washed, dried and sealed.

#### *Rhodopseudomonas spheroides*

*Rhodopseudomonas spheroides* was purchased from Hangzhou Lidong Company and enriched in Fannier liquid medium under the conditions of 30 °C and pH 7. The specific configuration of Fannier solution was 0.50 g/L K_2_HPO_4_, 1 g/L NH_4_Cl, 0.20 g/L MgCl_2_, 1 g/L NaCl, 5 g/L NaHCO_3_, 1 g/L yeast extract, 121 °C, sterilized for 30 min.

#### SRB

A self-detaching SRB (NCBI registration number is MT804386) with strong ability to metabolize SO_4_^2−^ was used for the experiment. SRB was inoculated in a modified Starkey-type medium and continuously enriched in a biochemical incubator (Shanghai Huitai Instrument Company, LRH-250) under the conditions of 35 °C and pH 7. The medium was changed every 5 days until H_2_S and black precipitate appeared^22^. The composition of the modified Starkey-type medium was 1 g/L yeast extract, 4 g/L ethanol, 0.10 g/L ascorbic acid, 0.50 g/L K_2_HPO_4_, 2 g/L MgSO_4_·7H_2_O, 0.50 g/L Na_2_SO_4_, 1 g/L NH_4_Cl, 0.10 g/L CaCl_2_·H_2_O, 1.20 g/L (NH_4_)_2_Fe(SO_4_)^2−^·6H_2_O, 121 °C, sterilized for 30 min. The drugs in this study were purchased from Tianjin Fuchen Company, all of which were Analytical reagent.

#### AMD

AMD is based on the simulated fabrication of the measured concentration of pollutants in AMD of a mining area in Huludao City, Liaoning Province, with pH 4, SO_4_^2−^, Cu^2+^, Zn^2+^, Ca^2+^ and Mg^2+^ concentrations of 816 mg/L, 10 mg/L, 20 mg/L, 100 mg/L and 50 mg/L respectively. AMD was prepared daily, 1L each time.

### Preparation of immobilized particles

According to the group's previous study^23^, SRB-LP and R-SRB-LP were prepared according to the optimal ratio (mass fraction of 3% lignite, 10% SRB) and (mass fraction of 3% lignite, 10% SRB, 10% *R. spheroides*) respectively. Firstly, 9% polyvinyl alcohol and 0.5% sodium alginate were dissolved in distilled water, kept under seal at room temperature for 24 h until sufficiently swollen and then stirred continuously in a constant temperature water bath (Hangzhou Hengyi instrument technology effective company, model HH-4) at 90 °C until no bubbles were produced. The lignite was then slowly added to the gel at a water bath temperature of 40 °C and fully stirred to uniform without bubbles. And then, the above materials were taken out and sealed in the room, and when cooled to 37 ± 1 °C, add the bacterial solution (SRB-LP with SRB concentrated solution only, R-SRB-LP with *R. spheroides* and SRB concentrated solution) and stirred well until no air bubbles. Then, the gel mixture was aspirated with a peristaltic pump (Shanghai Kachuaner Fluid Technology Company, model STP-F01A) and directly dropped into a 2% CaCl_2_ saturated boric acid solution at pH 6. The crosslinking was carried out with a magnetic stirrer (Jintan District Xicheng Xinrui instruments, model JJ-1A) at a stirring rate of 100 rpm and the immobilized particles were taken out after 4 h, the particle size is about 0.5 cm. Rinsed with 0.90% physiological saline, and then the surface water was aspirated, and this operation was repeated three times to form SRB-LP and R-SRB-LP, respectively. The particles were activated with a modified Starkey-type medium without organic components for 12 h in an anaerobic environment before use.

### Dynamic experiments

**Figure 1:**
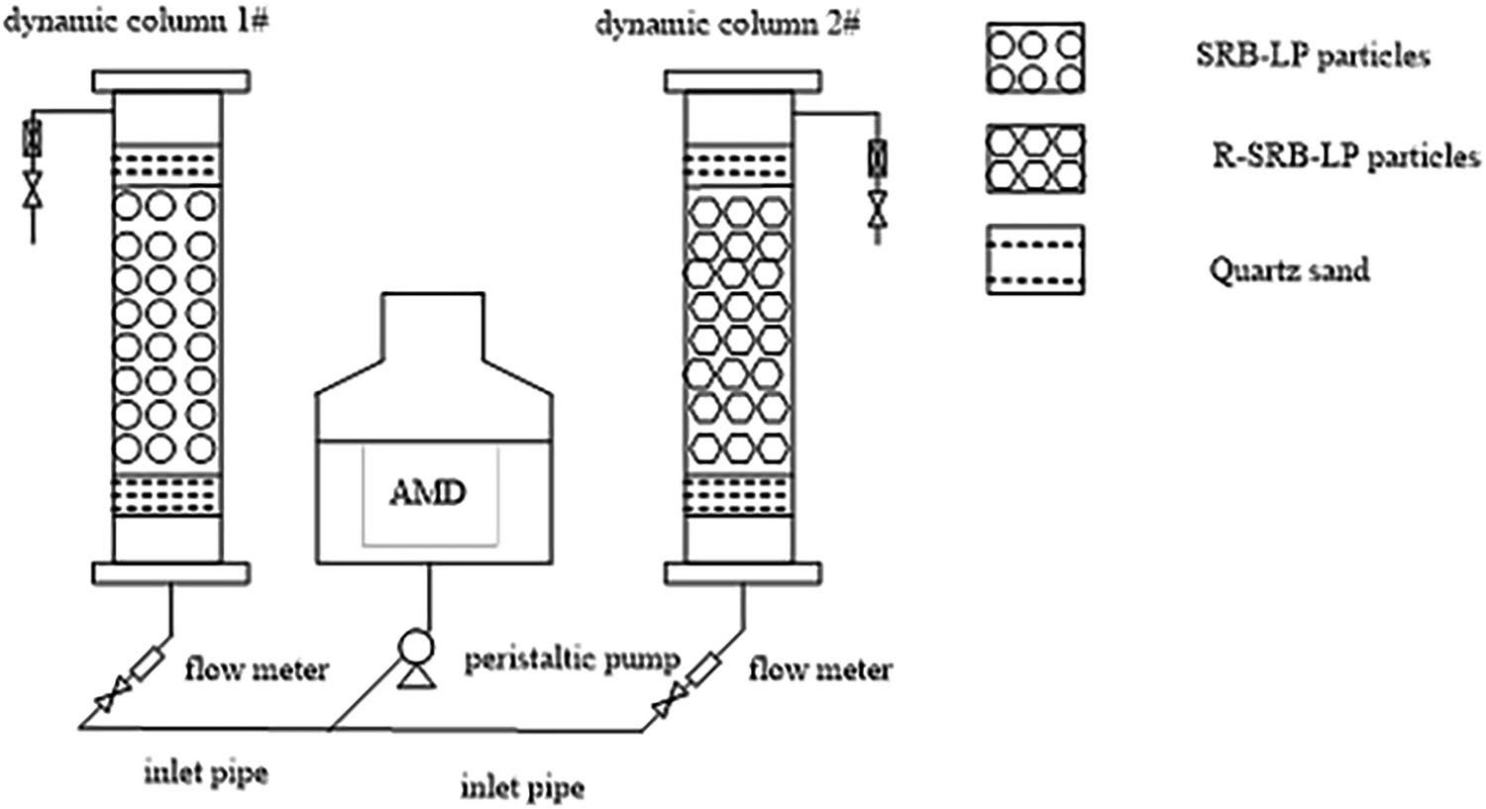
Experimental device.

The dynamic test device is shown in Fig. 1 and consists of two sets of dynamic columns with a diameter of 54 mm and a height of 500 mm.

The upper and lower layers were placed with 50 mm quartz sand with a particle size of 3–5 mm, the middle layer of column 1# was 250 mm SRB-LP, and the middle layer of column 2# was 250 mm R-SRB-LP. Column 1# was set as the blank group and column 2# as the experimental group. The inlet valve was opened and the AMD was continuously supplied from bottom to top at a flow rate of 0.398 mL/min. Three groups of experimental devices were set up, and the temperature was room temperature (25 °C) for 30 days. The water samples of influent water and effluent water from two dynamic columns were taken at 8am every day, and the water samples were filtered through a 0.45 μm filter membrane, then measured the concentrations of SO_4_^2−^, Zn^2+^, and Cu^2+^, pH, COD release and ORP value. The solution pH was measured by the glass electrode method (GB 6920-86), the concentration of SO_4_^2–^ was measured by the barium chromate spectrophotometric method (HJ/T 342-2007), the concentrations of Cu^2+^, Zn^2+^ were measured by flame atomic spectrophotometer method (GB/T 9723-2007), the ORP value was determined by the glycol electrode method (GB/T 9354-1999), and the COD was measured by the rapid extinction spectrophotometry method (HJ/T 399-2007). The removal efficiency was calculated using w = (C_0_ − C_1_)/C_0_ × 100%, where w represents the pollutant removal rate, %. C_0_ represents the initial pollutant concentration, mg/L. C_1_ represents the pollutant concentration after treatment, mg/L.

### SO_4_^2−^ reduction kinetics and Zn^2+^ adsorption kinetics

The SO_4_^2−^ reduction kinetics and Zn^2+^ adsorption kinetics were studied.

SO_4_^2−^ reduction kinetics experiment: 816 mg/L sulfate wastewater with pH of 4 was prepared. R-SRB-LP was added to the wastewater at the ratio of solid to liquid of 1:10, and continuous oscillation was carried out in a shaker at 30 °C and 150 r/min. The concentration of SO_4_^2−^ was measured at 1, 3, 5, 7, 9, 12, 18, 21, 24, 30, 36, 48, 72, 96, 120 h.

Zn^2+^ adsorption kinetics experiment: 20 mg/L Zn^2+^ with pH of 4 was prepared. R-SRB-LP was added to the wastewater at the ratio of solid to liquid of 1:10, and continuous oscillation was carried out in a shaker at 30 °C and 150 r/min. The concentration of Zn^2+^ was measured at 1, 3, 5, 7, 9, 12, 18, 21, 24, 30, 36, 48, 72, 96, 120 h.

### Reaction product characteristics

To explore the mechanism of AMD repaired by immobilized particles, the morphology of particles before and after reaction was characterized by JSM 7200F scanning electron microscope (SEM, produced in Japan), the surface functional groups of the particles before and after the reaction were analyzed by Niligao IN10 Fourier transform infrared spectrometer (FTIR, produced in USA), and the changes of the specific surface area of the particles before and after the reaction were analyzed by a Mac ASAP2460 analyzer (produced in USA) using N_2_ adsorption and desorption.

## Results and discussion

### ORP value analysis

**Figure 2:**
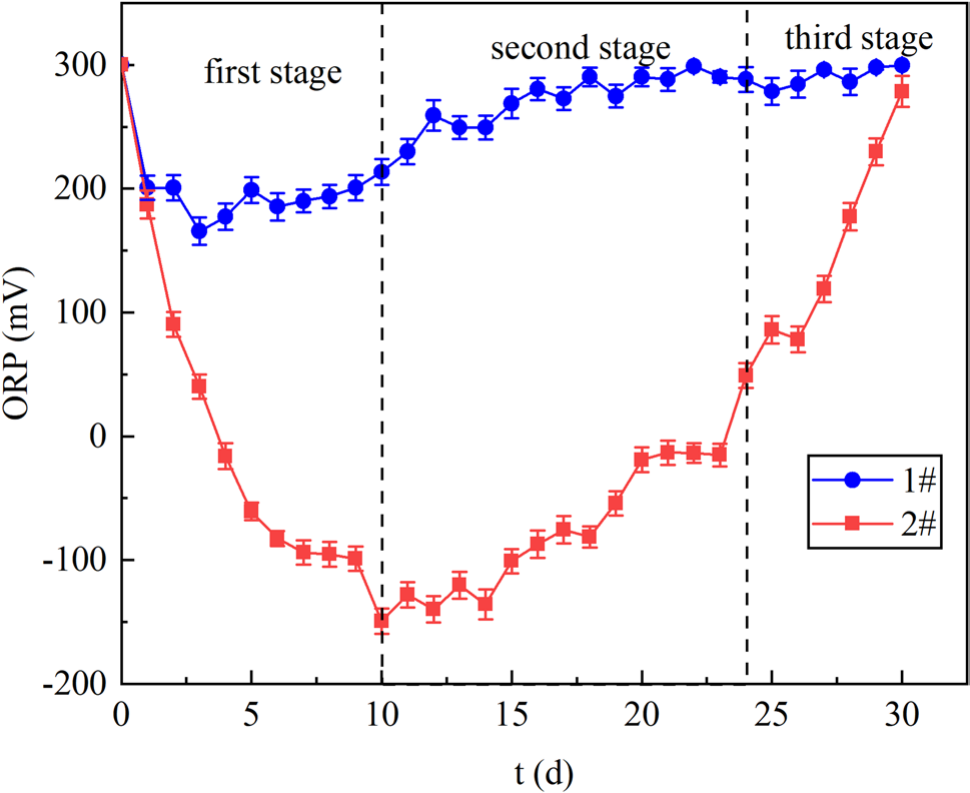
Changes of ORP values in dynamic columns 1# and 2#.

Figure 2 provides the changes of Oxidation Reduction Potential (ORP) values during the reaction. ORP values could initially reflect the strength of SRB activity. It was reported that SRB grows vigorously when the ORP value is from – 50 to − 300 mV^24^. The average ORP in column 1# was 246 mV. In the first stage, the ORP in column 1# was in the range of 200.78–174.71 mV, manifesting that SRB activity was low. SRB is a heterotrophic organism, and its metabolism needs the support of carbon source. At this stage, pH was suitable for SRB growth, the concentration of metal ions did not pose a threat to SRB activity, but the SRB activity was low, indicating that the organic matter in column 1# could not provide a large number of SRB growth, and the limited organic matter might come from the bacterial solution added in the preparation of particles. In the second and third stages, ORP increased from 288.33 mV to 299.73 mV, showing that the organic matter in the bacterial solution was fully utilized, and SRB was almost completely inactivated.

**Figure 3:**
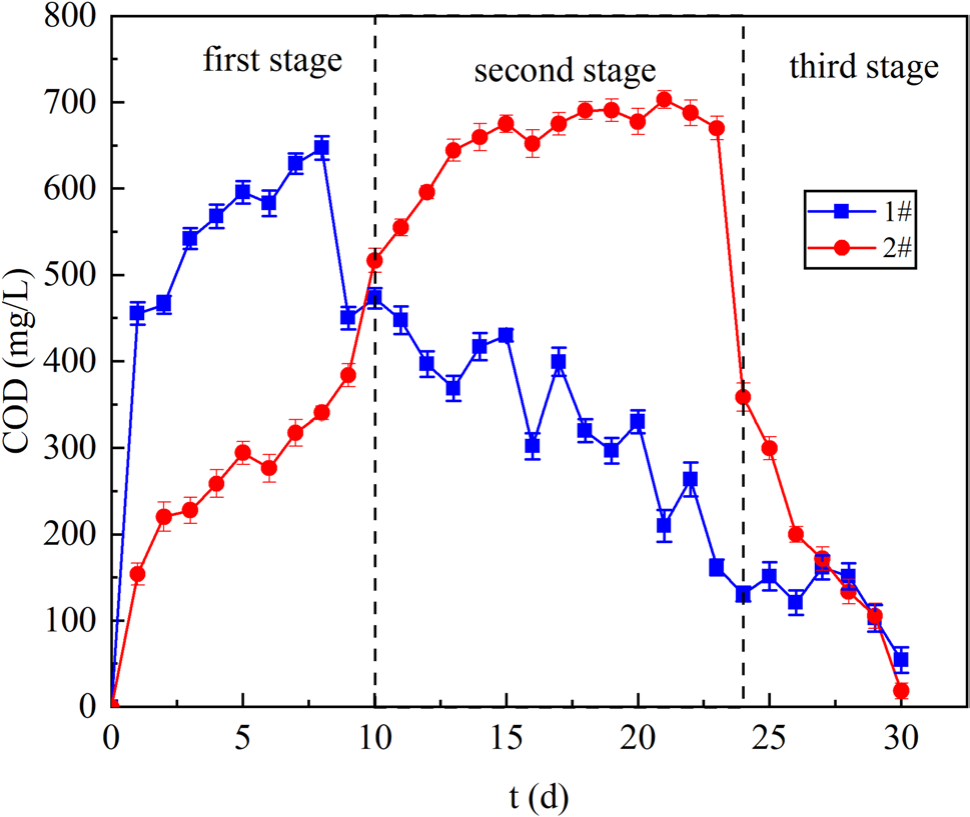
COD release in dynamic columns 1# and 2#.

Figure 3 shows that the curve of column 2# was below 1# and the ORP was in the range of − 50 mV to − 300 mV from day 5 to 19, which indicates that SRB growth in column 2# was better than that in column 1#. Under the condition of not adding any carbon source, column 1# maintained the activity of SRB for only 5 days with poor activity due to the lack of continuous carbon source supply. However, SRB in column 2# remained active and had relatively high activity. This proves that *R. spheroids* could degrade lignite to form small molecular organic matter and provide carbon source for SRB. In the first stage, ORP decreased from 187.16 mV to − 98.83 mV. It has been reported that SRB activity is related to pH, which is most suitable for SRB growth at 6–7.50^25^. At this stage, the pH of the solution gradually increased to this range, and the carbon source was sufficient, so the growth of SRB was getting better and better. In the second stage, ORP increased from − 149.14  to − 15.18 mV. At this stage, the carbon source was sufficient and the pH was suitable for SRB growth, but the activity of SRB decreased because of the inhibition of Cu^2+^ and Zn^2+^ on SRB. In the third stage, ORP increased from 49.03 mV to 278.60 mV. In this stage, the rate of SRB deactivation was accelerated due to low pH, toxicity of high concentrations of Cu^2+^ and Zn^2+^and shortage of carbon source.

### COD release analysis

Figure 3 shows the change of COD content in the effluent of columns 1# and 2#. SRB consumes COD in the process of SO_4_^2−^ reduction, Liu and Vossoughi et al.^26,27^ showed that the reduction of 1 g SO_4_^2−^ by SRB required 0.67 g COD. The average effluent COD from column 1# was 428.20 mg/L. The effluent COD increased from 423.62 to 646.72 mg/L in the first stage. Most of these COD came from the release of lignite^28^. From the 1# column ORP, it can be seen that the activity of SRB is poor due to the lack of carbon source at this stage, indicating that SRB cannot effectively utilize the organic matter released by lignite. In the second and the third stage, the effluent COD decreased from 472.99 to 54.01 mg/L. The capacity of lignite to release organic matter was limited, the leaching of COD was less and less, so the effluent COD declined.

The average effluent COD of column 2# was 428.20 mg/L. Kunlanit et al.^29^ indicated that COD was released when biomass materials were decomposed by anaerobic fermentation microorganisms, so the release of COD in column 2# was mainly attributed to the metabolic degradation of lignite by *R. spheroides*. In the first stage, the effluent COD of column 1# increased from 160.23 to 383.58 mg/L. *R. spheroides* was able to decompose lignite to provide carbon source for SRB growth. According to ORP, SRB growth gradually reached a vigorous state at this stage, and consumed more and more carbon source. While the effluent COD was growing at this time, which could indicate that the activity of *R. spheroides* was getting better and better. In the second stage, the effluent COD increased from 516.42 to 669.71 mg/L. From the 15th day, the effluent COD tended to be stable and gradually stabilized at about 680 mg/L. At this stage SRB was in a state of inhibition by metal ions, the carbon source consumed would be reduced. The COD remained flat indicating the ability to produce carbon source decreased in the system from day 18. This may be owing to diminished activity of *R. spheroids.* Quan^30^ et al. found that metal ions could inhibit the activity of *R. spheroides*. Due to the reduced ability of lignite to adsorb metal ions at this stage, metal ions accumulated in the system and caused toxic effects on *R. spheroids*. Although the ability of the system to produce carbon decreased from day 18, it was clear from curve 2# that sufficient carbon was produced to support the growth of SRB. In the third stage, the COD decreased from 358.03 to 18.25 mg/L. At this stage, the adsorption of heavy metals by lignite was saturated, and the accumulation of metal ions in the system increased, resulting in the gradual inactivation of *R. spheroides* and the reduction of carbon source production. Since the growth of SRB requires carbon source, the lack of a carbon source in the third stage was one of the factors contributing to SRB death.

### Analysis of pH enhancement effect

**Figure 4:**
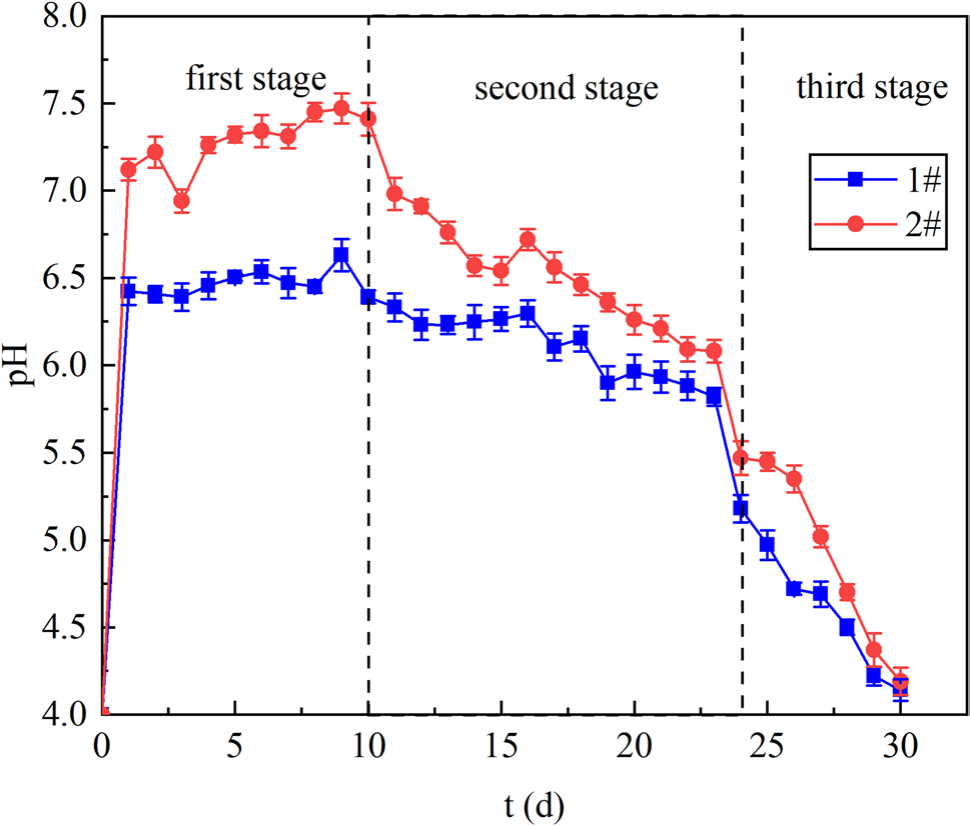
pH enhancement effect in dynamic columns 1# and 2#.

Figure 4 shows the effect of columns 1# and 2# on pH enhancement. The average pH of the effluent from column 1# was 5.88. In the first stage, the pH was in the range of 6.39–6.63. It can be seen from ORP and COD that SRB in column 1 # had almost no activity due to the support of no carbon source. Therefore, the slow increase of pH mainly depended on lignite, indicating that lignite has the ability to regulate pH, which is consistent with the results of Dong^31^. Lignite can release alkalinity to combine with H^+^ in solution to raise pH. In the second and third stage, the effluent pH decreased from 6.39 to 4.14. The decrease in pH was mainly due to the fact that the negative charge released by the lignite tended to saturate with the combination of H^+^, and the ability to regulate pH became weaker and weaker.

The average effluent pH of column 2# was 6.4 and the highest pH was 7.60. The effluent pH in the first stage was in the range of 7.24–7.60. The effluent pH of column 2# was higher than that of column 1 # because H^+^ was not only neutralized by alkaline substances released by lignite, due to the degradation of the lignite by *R. spheroides* continuously provided carbon source for the SRB and kept its activity, more H^+^ was consumed by the SRB as electron. The existence of a large number of active oxygen-containing functional groups on the lignite surface makes lignite negatively charged. The combination of H^+^ and negatively charged can improve the pH of the solution. From the 1 # column, the pH could be raised to about 6.50 only by lignite adsorption in the first stage, this range of pH was suitable for SRB survival, so lignite could remove the inhibition of acid on SRB and enhance SRB activity, which was consistent with ORP. At this stage, the adsorption capacity of lignite was strong, the SRB activity was enhanced and the consumption of H^+^ was increased, so the effect of pH improvement was good.

In the second stage, the effluent pH decreased from 7.11 to 6.20. At this stage, due to the limited negative charge contained in lignite, the removal effect of H^+^ decreased. However, from the 1# column, it can be seen that the pH could be increased to about 6 by lignite adsorption. It was in the suitable growth range of SRB, the existence of lignite could also maintain the stability of pH system. According to ORP and SO_4_^2−^, the activity of SRB showed a downward trend, which was caused by the inhibition of metal ions on SRB. As SRB activity decreased, less and less H^+^ could be consumed as electrons by SRB, and the alkaline released by lignite was gradually neutralized by H^+^, the effect of pH improvement was getting worse and worse from the second stage.

In the third stage, the effluent pH decreased from 5.61 to 4.35. As the neutralisation of H^+^ by lignite at this stage tended to saturate, it gradually lost its ability to raise pH, making the pH system and its instability. Under acidic conditions (pH < 5), high concentrations of protons would lead to high diffusion pressure in the cell membrane of microorganisms^32^, more energy is used to pull protons out of the membrane through proton motive force to maintain the internal pH value, less energy is used for cell growth^33^, the pH at this stage affected the SRB activity. Due to the reduced of H^+^ neutralization capacity of lignite and the reduced of electron consumption caused by SRB inactivation, the pH in the system decreased.

### Analysis of SO_4_^2−^ removal effect

**Figure 5:**
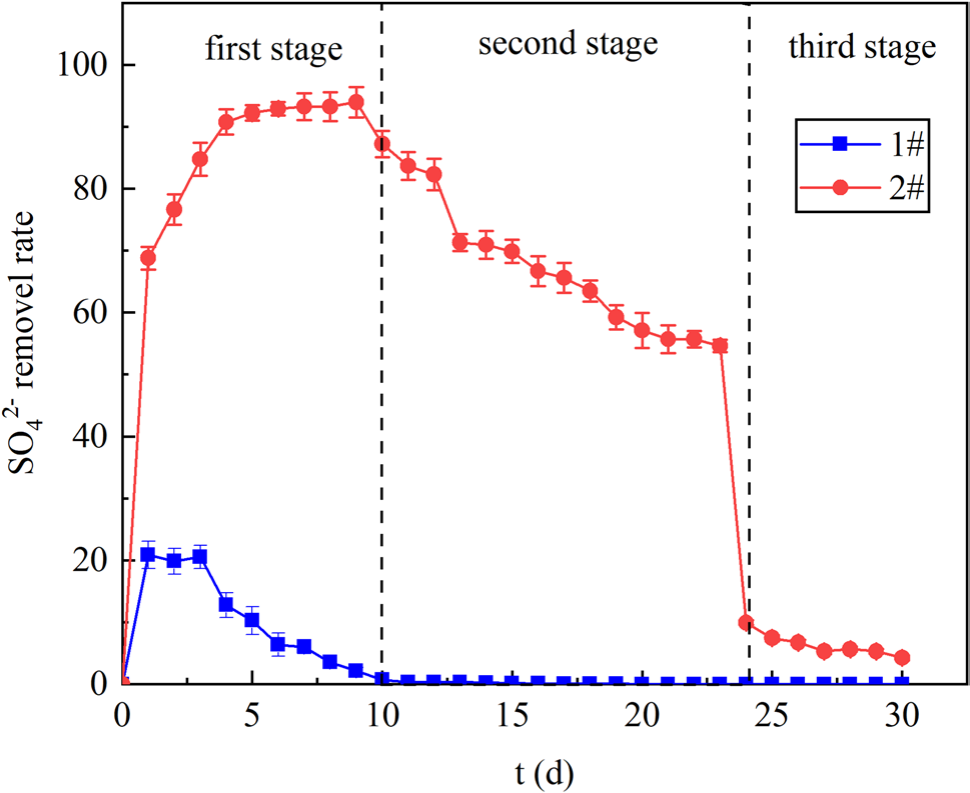
Removal rate of SO_4_^2-^ in dynamic columns 1# and 2#.

Figure 5 shows the effect of SO_4_^2−^ removal by 1# and 2# dynamic columns. Our previous experiment^28^ showed that lignite had no ability to remove SO_4_^2−^, so the removal of SO_4_^2−^ by the system mainly depended on the reduction of SRB. SO_4_^2−^ can also react to the activity of SRB. The average SO_4_^2−^ removal rate of column 1# was 3.61%. In the first stage, the removal rate of SO_4_^2−^ decreased from 21.39% to 0. Due to the absence of the carbon source in column 1#, the SRB only remained active for a few days and could last only a few days, so the SO_4_^2−^ reduced by SRB through anaerobic reduction was less. The removal of SO_4_^2−^ in the second and third stages was 0, which would also indicate that the SRB was deactivated at this stage, in line with the findings of the ORP value stage.

The average removal rate of SO_4_^2−^ in column 2# was 59.08% and the highest removal rate was 93.97%. In the first stage, the SO_4_^2−^ removal rate increased from 68.79 to 93.97%. At this stage, as the environment of the reaction system gradually reached the SRB comfort conditions, the SRB activity gradually raised, so the SO_4_^2−^ removal rate gradually increased. In the second stage, the removal rate of SO_4_^2−^ decreased from 87.23 to 54.61%. Although the activity of the SRB remained good at this stage, due to the inhibition of metal ions, the activity of SRB was affected, so the removal rate of SO_4_^2−^ decreased. Moreover, metal sulfides generated by metal ions and S^2−^ were adsorbed on the surface by the lignite, which would encrust microorganism, restricting mass transfer between reactants and microbial enzymes, and ultimately making the removal of sulfate less effective^34^. In the third stage, the removal rate of SO_4_^2−^ decreased from 9.93 to 4.26%. Owing to the lack of carbon source in the system, pH decreased and metal ions accumulated had toxic effects on SRB, SRB gradually lost its activity, so the removal rate of SO_4_^2−^ was low.

### Analysis of Cu^2 +^, Zn^2 +^ removal effects

**Figure 6:**
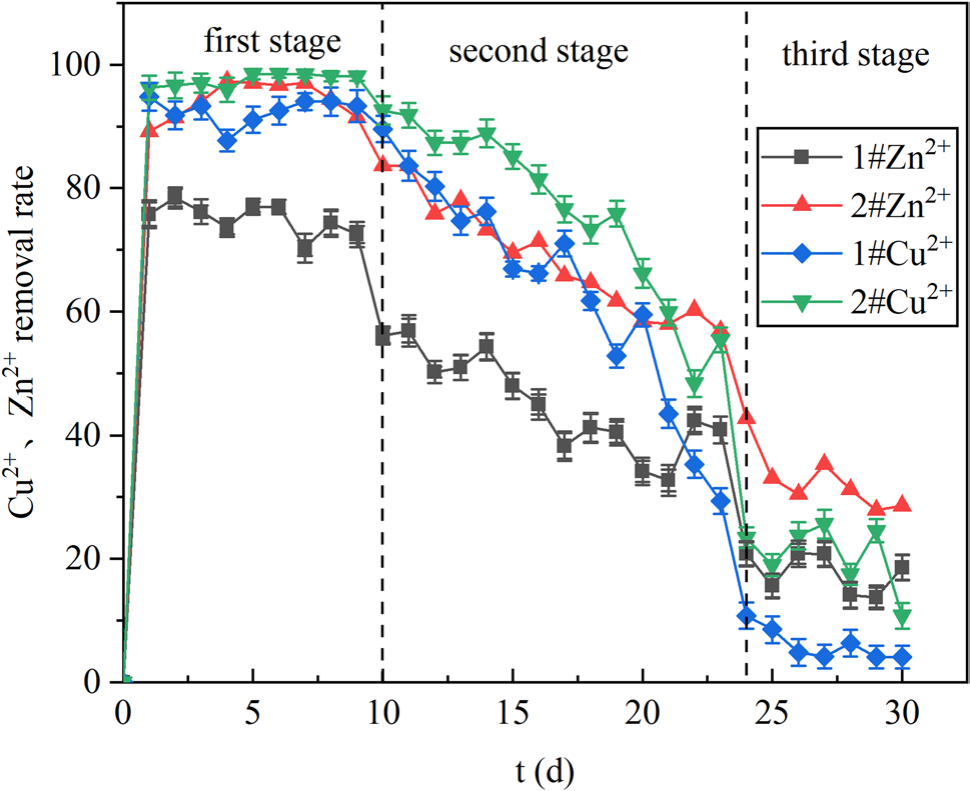
Cu^2+^ and Zn^2+^ removal effects in dynamic columns 1# and 2#.

Figure 6 shows the effect of columns 1# and 2# on the removal of Cu^2+^ and Zn^2+^. The average removal rates of Cu^2+^ and Zn^2+^ by column 1# were 58.87% and 47.05% respectively. In the first stage, the removal rate of Cu^2+^ by column 1# was 94.80%–89.59% and the removal rate of Zn^2+^ was 75.76%–72.49%. The removal of metal ions at this stage mainly depended on the adsorption of lignite, which has a large number of aerobic functional groups on its surface, and these functional groups can chelate with metal ions to adsorb them on the surface^35^. Moreover, according to K_sp_Cu(OH)_2_ = 2.2 × 10^–20^ and K_sp_Zn(OH)_2_ = 1.2 × 10^–17^, some of the metal ions were removed in the form of hydroxide as the pH increased^36^. In the third stage, the removal efficiency of Cu^2+^ declined from 83.64 to 10.78% and that of Zn^2+^ from 56.13 to 13.75%. In the middle and late stages of the reaction, the adsorption sites on the lignite surface gradually reduced until the adsorption saturation, and the removal effect of Cu^2+^ and Zn^2+^ continued to decrease.

The average removal rates of Cu^2+^ and Zn^2+^ by column 2# were 69.76% and 67.91%, respectively, and the highest removal rates were 98.52% and 97.03%. In the first stage, the removal efficiency of Cu^2+^ was 96.28–98.52%, and the removal efficiency of Zn^2+^ was 89.22–94.42%. The removal of Cu^2+^ and Zn^2+^ was stronger in column 2# than in column 1# because the biological removal of metal ions was present in column 2# in addition to the adsorption of lignite. *R. spheroides* can destroyed the structure of lignite and increased the adsorption sites of lignite to adsorb more metal ions^37^. Li et al.^38^ found that the effluent S^2−^ was less than 1 mg/L at pH above 6.5 during the effect of pH on S^2−^ in the AMD treatment test, indicating that the precipitation of sulfate ions with metal ions was easy at higher pH conditions. According to the pH, in the first stage, the pH could be increased to about 7.3 by the synergistic effect of lignite and SRB. Therefore, Zn^2+^ and Cu^2+^ in the system could be combined with reduced S^2−^ and removed in the form of CuS and ZnS^39^. At the same time, SRB could adhere to the surface of lignite to form biofilm, and a large number of extracellular polymers (EPS) were generated. Beech et al.^40^ found that bacteria themselves promote the binding of metals to S^2−^ when there was less metal sulfide in solution, cell walls and extracellular polymer (EPS) were able to bind metals thus accelerating solid formation. Therefore, the removal of Cu^2+^ and Zn^2+^ by column 2# at this stage was effective.

In the second stage, the removal efficiency of column 2# decreased from 92.57% to 55.39% for Cu^2+^ and from 83.64% to 55.39% for Zn^2+^. Kieu et al.^41^ indicated that 6.0–7.5 mg/L heavy metal ion concentration could inhibit the growth of SRB; above 7.5 mg/L heavy metal ion concentration was the toxic concentration that could lead to SRB death. In this stage, due to the limited adsorption sites on the surface of lignite, the adsorption properties of lignite for Cu^2+^ and Zn^2+^ gradually decreased, and the metal ions gradually accumulated. Although the carbon source was sufficient at this stage, the pH could be increased to about 6.50 under the combined action of lignite and SRB, which would not have a great impact on the activity of SRB and the formation of metal sulfate. The activity of SRB was reduced by the inhibition and poison of metal ions. The adsorption of metal ions by lignite decreased, and at the same time, the weakened activity of SRB resulted the reduced S^2-^ reduced, the generated metal sulfides declined, so the removal efficiency of Cu^2+^ and Zn^2+^ decreased.

In the third stage, the removal efficiency of Cu^2+^ decreased from 23.42% to 10.78% and the removal efficiency of Zn^2+^ decreased from 42.75% to 28.62%. At this stage, the adsorption performance of lignite tended to be saturated, and the adsorbed metal ions decreased. The accumulation of metal ions had a more serious toxic effect on SRB. So, this stage of SRB due to pH and metal toxicity, lack of carbon source, SRB activity decreased, S^2-^ reduced. At the same time, it can be seen from pH that lignite cannot maintain the stability of pH system at this stage, which affected the formation of metal sulfides. So, the removal efficiency of Cu^2+^ and Zn^2+^ by 2# column was low.

### Reduction kinetics of SO_4_^2−^ and adsorption kinetics of Zn^2+^

Kinetic studies are crucial for determining the dynamic reduction (adsorption) rate and controlling the reduction (adsorption) mechanism. Zero-order kinetics, first-order kinetics, pseudo-first-order kinetics and pseudo-second-order kinetics were used to simulate the relationship between reduction (adsorption) rate and time of SO_4_^2-^, and Zn^2+^, respectively.

1 $${\text{C}}_{{\text{t}}} = {\text{C}}_{{0}} - {\text{k}}_{{0}} {\text{t}}$$

2 $${\text{ln C}}_{{\text{t}}} = {\text{ ln C}}_{{0}} - {\text{ k}}_{{1}} {\text{ t}}$$

3 $${\text{ln(q}}_{{\text{e}}} - {\text{ q}}_{{\text{t}}} {\text{ ) = lnq}}_{{\text{t}}} \, - {\text{ k}}_{{2}} {\text{ t}}$$

4 $$\frac{t}{{q_{t} }} = \frac{1}{{k_{2} q_{e}^{2} }} + \frac{t}{{q_{e} }}$$

In the formula, C_t_ represents the concentration of SO_4_^2−^ at time t (h), mg/L. C_0_ represents initial SO_4_^2−^ concentration, mg/L. k_0_ represents zero-order reaction rate constant, mg·L^−1^·h^−1^. k_1_ represents the first-order-reaction rate constant, h^−1^. q_e_ is the adsorption amount at adsorption equilibrium, mg g^−1^. q_t_ is the adsorption amount when the adsorption time is time t (min), mg g^−1^. k_3_ is the pseudo-first-order kinetic reaction rate constant, min^−1^. k_4_ is the pseudo-second-order kinetic reaction rate constant, g (mg^−1^ min^−1^).

**Figure 7:**
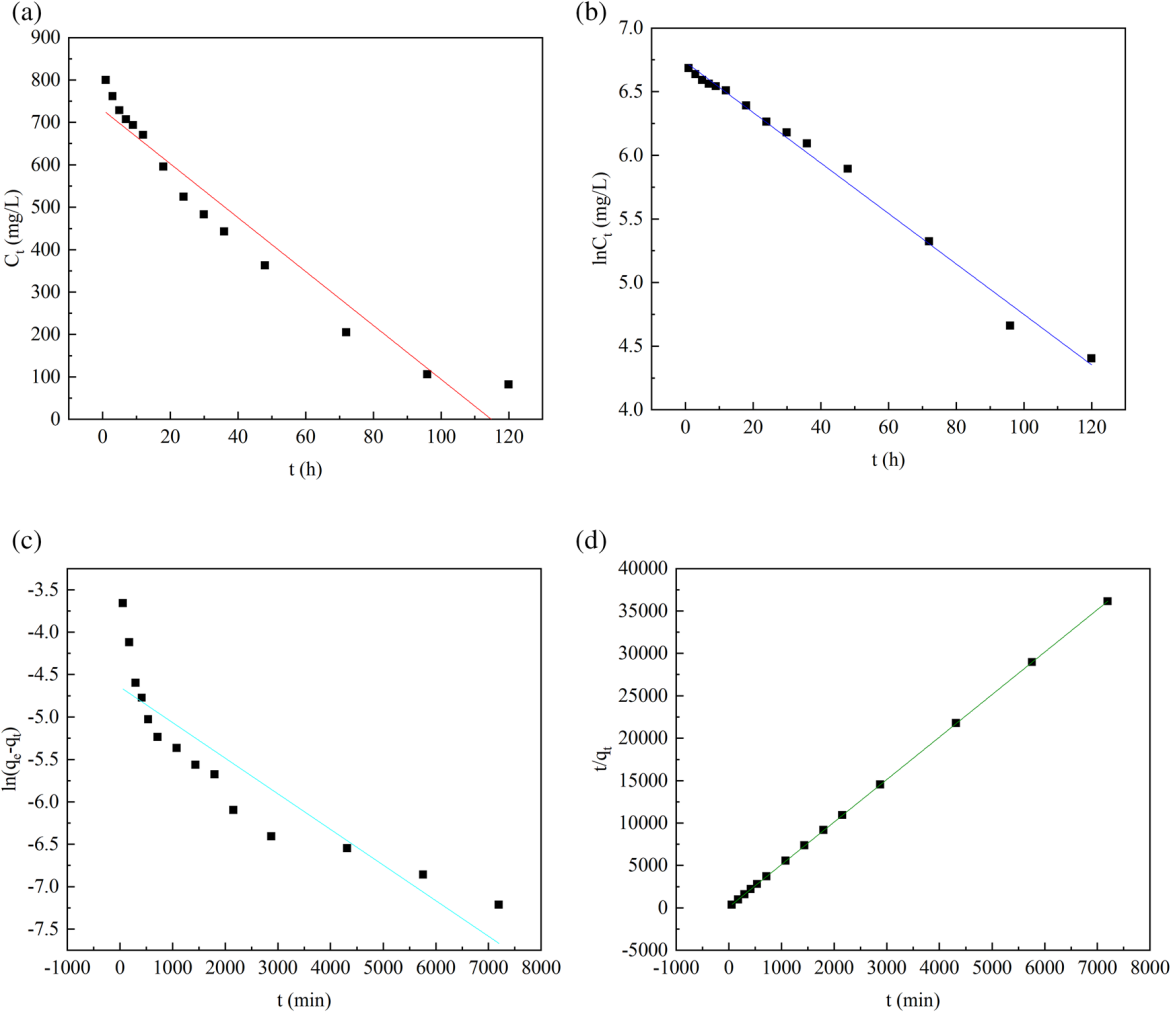
Kinetic fitting. (**a**) Zero-order kinetics of reduction of SO_4_^2−^. (**b**) First-order kinetics of SO_4_^2−^. (**c**) Pseudo-first-order kinetics of Zn^2+^. (**d**) Pseudo-second-order kinetics of Zn^2+^.

The kinetic fitting diagram is shown in Fig. 7. (a) As the zero-order kinetic fitting of SO_4_^2-^ reduction. (b) As the first-order kinetic fitting of SO_4_^2-^. (c) As the pseudo-first-order kinetic fitting of Zn^2+^, and (d) as the pseudo-second-order kinetic fitting of Zn^2+^. According to the linear fitting results, the dynamic parameters and the correlation coefficient of the evaluation are shown in Table 1.

**Table. 1:** SO_4_^2−^ Zero-order kinetics, first-order kinetics parameters and Zn^2+^ pseudo-first-order kinetics, pseudo-second-order kinetics parameters.

Object	Zero-order		First-order		Object	Pseudo-first-order		Pseudo-second-order	
Reduction kinetics of SO_4_^2-^	k_0_ (mg L^−1^ h^−1^)	R^2^	k_1_ (h^−1^)	R^2^	Adsorption kinetics of Zn^2+^	k_2_	R^2^	k_3_	R^2^
	6.359	0.941	0.0198	0.991		0.0042	0.797	0.268	0.999

Obviously, in the reduction kinetics of SO_4_^2−^, R^2^ (0.991) of the first-order kinetics was greater than R^2^ (0.941) of the zero-order kinetics, indicating that the first-order reaction kinetics model was more suitable for the reduction process of SO_4_^2−^ by SRB in R-SRB-PL. The reduction of SO_4_^2−^ is mainly affected by electron acceptors, and the main removal process of SO_4_^2−^ in wastewater is SRB alienation reduction. In the adsorption kinetics of Zn^2+^, the pseudo-second-order kinetic R^2^ is greater than the pseudo-first-order kinetic R^2^. It is indicated that pseudo-second-order kinetics is more suitable for the adsorption process of metal ions by R-SRB-PL, and electron sharing or electron transfer may exist in the adsorption process of metal ions by R-SRB-PL.

### SEM characterization analysis

**Figure 8:**
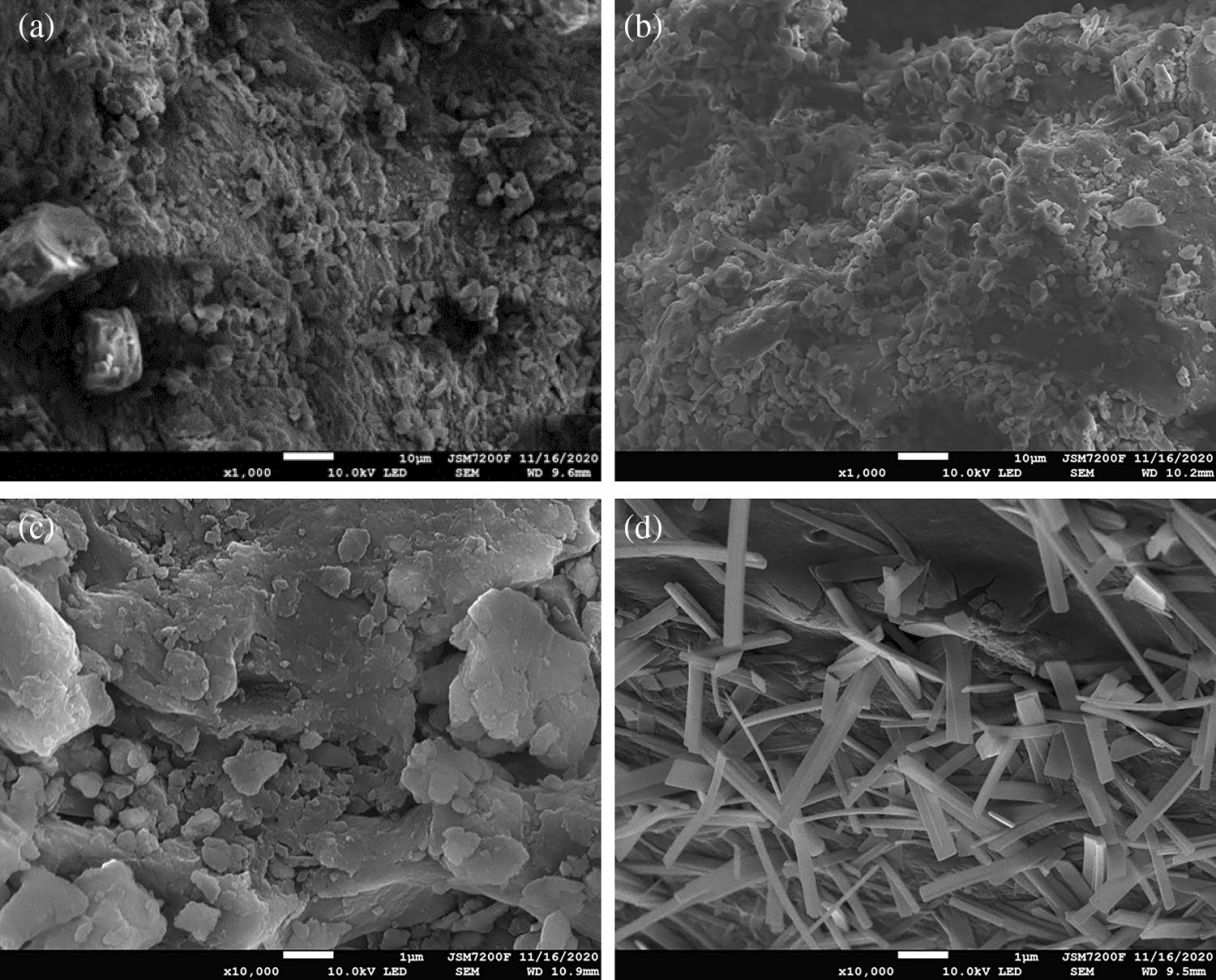
SEM images of SRB-LP and R-SRB-LP. (**a**) SEM image of SRB-LP before AMD treatment. (**b**) SEM image of SRB-LP after AMD treatment. (**c**) SEM image of R-SRB-LP particles before AMD treatment. (**d**) SEM image of R-SRB-LP after AMD treatment.

The results of the SEM are shown in Fig. 8. At the end of the dynamic experiment, SRB-LP and R-SRB-LP were dried, and the changes of particle morphology before and after the reaction were explored by SEM scanning. There was no significant change in the surface state of lignite SRB-LP, indicating that SRB alone could not degrade lignite. After R-SRB-LP reaction, a large number of cracks and pores appeared on the surface of lignite, and the organic matter that could not be degraded by *R. spheroides* was precipitated on the surface of lignite. These results illustrated that *R. spheroides* could destroy the structure of lignite, and enhanced the adsorption capacity.

### FTIR analysis

**Figure 9:**
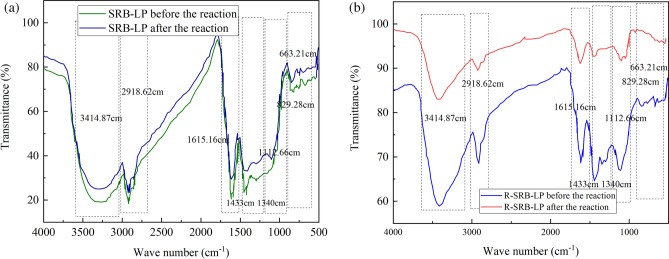
FTIR plots of SRB-LP and R-SRB-LP before and after AMD treatment. (**a**) FTIR of SRB-LP before and after reaction. (**b**) FTIR of SRB-LP before and after reaction.

In order to further study the changes of surface functional groups of lignite in SRB-LP and R-SRB-LP, the SRB-LP particles and R-SRB-LP particles before and after the reaction were detected by FTIR, and the results are shown in Fig. 9. The peak at 3414.87 cm^−1^ was attributed to the stretching vibration of enol-OH or N–H^42,43^. The position of 2918.62 cm^−1^ belonged to the antisymmetric stretching vibration of alkanes C–H. The peak at 1615.16 cm^−1^ was benzene C=C stretching vibration^44^. The position peaks of 1433 cm^−1^ and 1340 cm^−1^ were –CH_3_ asymmetry peak, CH_2_ planar bending peak or benzene skeleton vibration peak^45^. 1112.66 cm^−1^ position is assigned to the saturated C–O stretching vibration peak in tertiary alcohols. Peaks at 829.28 cm^−1^, 663.21 cm^−1^ represented the stretching vibrations of C–H substituents in the benzene ring structure.

It can be seen from Fig. 9a that in SRB-LP, the peak position only changed slightly, indicating that the structure of lignite in SRB-LP was not destroyed in a wide range. The reduction of peak positions around 3414.87 cm^−1^, 2918.62 cm^−1^, 1615.16 cm^−1^, 1112.66 cm^−1^, 829.28 cm^−1^, 663.21 cm^−1^ may be due to the replacement of some functional groups in the lignite by metal ions with H^+^ throughout the process, resulting in a reduction of some functional groups and leading to a reduction in peak positions.

It can be seen from Fig. 9b that in R-SRB-LP, the peak position changed greatly, not only the peak decreased but also the peak disappeared, which indicated that the lignite structure in R-SRB-LP was destroyed. The peak intensity at 3414.87 cm^−1^ and 1615.16 cm^−1^ decreased, indicating that during the degradation of lignite by *R. spheroides*, the structure of-OH and benzene ring in lignite structure was destroyed. The decrease in the vicinity of 2918.62 cm^−1^ indicated that *R. spheroides* could break the C–C bond in the molecular structure of lignite, thus breaking the side chains of aromatic rings, some alkanes and olefins in the lignite structure. The disappearance of peaks at 829.28 cm^−1^, 663.21 cm^−1^ was attributed to the substitution effect of metal cations.

Due to the absence of *R. spheroides* in SRB-LP, there was little difference in the peak position before and after the reaction, while there was *R. spheroides* in R-SRB-LP, the difference of peak position before and after reaction was large and the peak disappeared. This shows that *R. spheroides* can decompose lignite and destroy lignite structure.

### Specific surface area analysis

Specific surface areas of SRB-LP and R-SRB-LP are compared as shown in Table 2. As can be seen from Table 1, the BET specific surface area and Langmuir specific surface area did not change much after the SRB-LP reaction. However, the BET specific surface area of R-SRB-LP after reaction was 1.58 times that before the reaction and the Langmuir specific surface was 1.53 times that before the reaction. This indicated that *R. spheroides* changed the surface structure of lignite. Some functional groups were dissolved, thereby increasing the number of voids on the surface of lignite. The specific surface area increased, and the adsorption performance of lignite was improved.

**Table 2:** Specific surface area analysis.

	BET specific surface area (m^2^/g)	BET correlation coefficient	Langmuir specific surface area (m^2^/g)	Langmuir correlation coefficient
SRB-LP before the reaction	6.8992	0.9990	15.392	0.9860
SRB-LP after the reaction	6.9012	0.9990	15.426	0.9860
R-SRB-LP before the reaction	7.4621	0.9990	16.6545	0.9860
R-SRB-LP after the reaction	11.8146	0.9990	25.5604	0.9860

**Figure 10:**
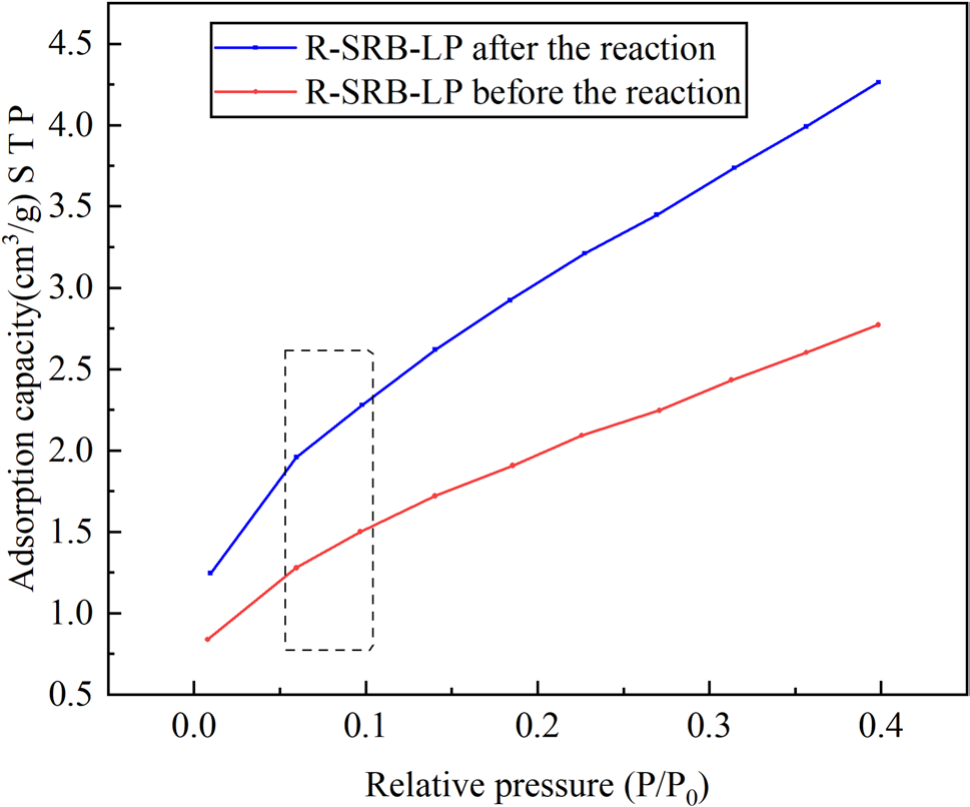
R-SRB-LP adsorption equilibrium isotherm conclusion.

Figure 10 shows the adsorption amount of R-SRB-LP versus relative pressure obtained from the test results. This adsorption equilibrium isotherm was consistent with the type II isotherm, which indicated that the adsorption process of lignite on metal ions was a free multilayer reversible adsorption process. The steep points of this isotherm are between 0.05 and 0.10, indicating the saturated adsorption amount of single molecule, which was equivalent to the completion of single molecule layer adsorption. The adsorption curve of R-SRB-LP particles after reaction was always above before reaction, which also proved that the adsorption performance of R-SRB-LP particles after reaction was better than that before reaction.

## Conclusions

By constructing 1# and 2# dynamic columns filled with SRB-LP and R-SRB-LP, the feasibility of decomposing lignite by *R. spheroides* as SRB carbon source and the treatment effect of R-SRB-LP on AMD were discussed. It is proved that R-SRB-LP treatment of AMD not only realizes the economy and sustainability of carbon source, but also resists the influence of external factors such as pH and heavy metals, and has good repair effect on AMD. R-SRB-LP repair AMD by lignite adsorption capacity and SRB anaerobic reduction, it has certain application value in dealing with a large number of AMD.

1. The test results showed that the SRB activity in column 1# was weak and the survival time was short because there was no continuous supply of carbon source. Under the same conditions, the SRB activity in column 2# was good and the survival time was long, indicating that the combination of *R. spheroides* and lignite could continuously provide carbon source for SRB.
2. The highest removal efficiencies of SO_4_^2−^, Cu^2+^ and Zn^2+^ by 2# column were 93.97%, 98.52% and 94.42%, respectively, and the highest pH value was 7.60, indicating that R-SRB-LP can resist the influence of external environment on SRB, so it has good repair effect on AMD.
3. SEM, FTIR and low temperature nitrogen adsorption characterization results showed that the lignite state of SRB-LP particles after reaction was similar to that before reaction. After the reaction of R-SRB-LP particles, the surface structure of lignite was destroyed, and the functional groups such as –OH and large benzene ring structure in lignite were broken, and the specific surface area after reaction was 1.58 times that before reaction. It was proved that *R. spheroides* provided carbon source for SRB by degrading lignite, and improved the adsorption capacity of lignite.
